# Effective analysis of job satisfaction among medical staff in Chinese public hospitals: a random forest model

**DOI:** 10.3389/fpubh.2024.1357709

**Published:** 2024-04-18

**Authors:** Chengcheng Li, Xuehui Meng

**Affiliations:** Department of Health Service Management, Humanities and Management School, Zhejiang Chinese Medical University, Hangzhou, China

**Keywords:** medical staff, job satisfaction, large public hospitals, random forest model, key associated factors

## Abstract

**Objective:**

This study explored the factors and influence degree of job satisfaction among medical staff in Chinese public hospitals by constructing the optimal discriminant model.

**Methods:**

The participant sample is based on the service volume of 12,405 officially appointed medical staff from different departments of 16 public hospitals for three consecutive years from 2017 to 2019. All medical staff (doctors, nurses, administrative personnel) invited to participate in the survey for the current year will no longer repeat their participation. The importance of all associated factors and the optimal evaluation model has been calculated.

**Results:**

The overall job satisfaction of medical staff is 25.62%. The most important factors affecting medical staff satisfaction are: Value staff opinions (Q10), Get recognition for your work (Q11), Democracy (Q9), and Performance Evaluation Satisfaction (Q5). The random forest model is the best evaluation model for medical staff satisfaction, and its prediction accuracy is higher than other similar models.

**Conclusion:**

The improvement of medical staff job satisfaction is significantly related to the improvement of democracy, recognition of work, and increased employee performance. It has shown that improving these five key variables can maximize the job satisfaction and motivation of medical staff. The random forest model can maximize the accuracy and effectiveness of similar research.

## Background

The loss and lack of medical staff is becoming a global problem (1). It is crucial to improve the quality of life and health of patients through a large and stable medical service team (2). The dense urban population has accelerated the spread of respiratory infectious diseases such as COVID-19, posing new challenges to the prevention and control of public health emergencies (3, 4). Indeed, The rapid realization of urban–rural integration also requires more medical services and support in China (5). Due to the continuous prevalence of the virus and the unsafe medical environment, medical workforce continue to drain in the post-epidemic era (6). The aging of the population and the decline of the birth rate have to some extent exacerbated the shortage of medical labor force in China (7). Meanwhile, Rural and urban areas have gradually become a medical service community with the implementation of graded diagnosis and treatment in a certain geographical range in China (8). High-quality medical and health resources will sink and radiate to surrounding areas through central cities (9). Therefore, this is a priority to retain public medical staff, especially the urban medical workforce.

Since the reform of China’s medical and health system in 2009, which established and improved the basic healthcare system covering urban and rural residents. China has expanded the coverage of basic medical insurance, implemented the reform of Diagnosis Related Groups (DRG) payment method and implemented the centralized purchase of drugs (10–12). The coverage of medical services has been continuously improved and the difficulty of seek medical services and cost of personal medical treatment have been effectively alleviated. It is easier for people to obtain safe, effective, convenient and inexpensive medical and health services along with the informatization construction of the hospital. Some scholars have systematically evaluated the satisfaction of burn patients and further found that the quality of care of medical workers can significantly improve patient satisfaction (13). Zhou et al. explored the association between patient satisfaction and nursing compliance and trust of medical workers of Chinese hypertensive patients (14). Moreover, Li et al. confirmed that the medical service quality of medical staff is the main factor affecting patient satisfaction from the perspective of inpatients (15). Of course, the implementation of a series of health reform policies not only reduces the economic burden of patients, but also greatly affects the income level of medical staff (16–18). The diversification of individual medical service needs is also increasing the daily workload of medical staff. At the same time, the demand for high-quality medical services has also increased the work difficulty of medical workforce (19, 20). Some studies have found that poor working environment and large workload will aggravate the job burnout of medical staff, and lead to resignation (21, 22). On the contrary, medical staff with higher job satisfaction tend to provide higher quality medical services and can effectively avoid medical accidents (23, 24). The current studies are more biased toward the patient’s medical experience and thus ignores the work experience of medical staff (25). Therefore, this is of great significance for improving the accessibility of medical services and maintaining social stability by exploring the current situation and associated factors of staff job satisfaction in public hospitals in China.

Building an optimal calculation model is the prerequisite for influencing factor analysis, which is crucial to ensure the robustness and accuracy of the analysis results. Multiple logistic regression models are widely used in related research due to their relatively simple theoretical assumptions (26). Regression models have greater advantages over OLS models in probability prediction (27). Li et al. analyzed the influencing factors of patient satisfaction through a multiple logistic regression model (28). Zhou et al. used a multi-level logistic regression method to test the associated factors of job satisfaction among medical personnel in 2018 (29). As one of the most widely used linear regression analysis models, the accuracy of the analysis results obtained by multiple regression models remains to be discussed due to the possible natural defects of collinearity sensitivity between independent variables. And then, logical regression models cannot properly handle massive multi-category variables. Hence, it is necessary to introduce discriminant analysis models into current research. The naive Bayesian algorithm is based on the posterior probability thinking of classical mathematical theory to establish models, greatly optimizing the complexity of traditional Bayesian algorithms in the calculation process (30). The discriminant algorithm logic assumes that the attributes of the dataset are independent of each other, which exhibits strong stability and consistency for different datasets. Bai et al. used naive Bayesian models to accurately classify different water sources subject to weather interference in the environmental field (31). Some scholars predicted that the physical behavior of patients with COVID-19 by using the naive Bayesian model (32). similarly, the random forest algorithm has gradually become a widely recognized classification algorithm by combining classification tree models. Random forest model have strong adaptability because of Strong adaptability (33). Random forest model reduce the risk of over fitting in the calculation process by improving the generalization ability. Some scholars have applied random forest models to studies on disease risk assessment, tumor diagnosis, and postoperative prognosis (34–36). Other scholars have explored disease risk prediction, diagnosis, and classification through random forest models (37–39). Compared to traditional methods, these two classification algorithms are praised as one of the best currently available algorithms, which are not susceptible to environmental noise and can well predict ample of explanatory variables.

In recent years, K-Nearest Neighbor (KNN) algorithms have gradually been widely used in different fields. Some scholars have conducted study on the prevention and control of agricultural diseases and insect pests through the KNN algorithm for disease identification (40). Other scholars have explored the use of KNN algorithms for disease prediction in the medical field (41). KNN algorithm, a Simple Classification Algorithm, does not need to estimate parameters, but the calculation amount is relatively large when the heterogeneity between samples is large. Meanwhile, the Gradient Boosting Decision Tree (GBDT) algorithm can optimize the model by using an additive model and a forward step algorithm (42). Some scholars have used GBDT algorithm to effectively predict the employability of graduates in the internship environment (43). A European study effectively predicted the impact of psychosocial factors on quality of life in older adults people through machine learning algorithms (44). Machine learning algorithms are being used by more and more scholars in the field of public health. Unfortunately, there are few studies exploring the optimal evaluation model for medical staff job satisfaction. Therefore, we attempt to incorporate the above algorithms into model comparisons in order to obtain more accurate analysis results.

This paper aims to explore the associated factors and best evaluation models of staff job satisfaction in Chinese public hospitals. And we attempt to identify strategies to improve job satisfaction among public medical staff based on empirical research results.

## Sample and methods

### Ethics statement

This study was approved and supported by the Zhejiang Provincial Health Commission, and the investigation was conducted after obtaining the consent and support of the relevant heads of 16 hospitals. All participating medical staff signed an informed consent form before filling out the questionnaire. This job satisfaction survey is anonymous and the content filled in is completely confidential.

### Study design and samples

The survey was conducted from December 12, 2017 to January 13, 2020, involving 16 provincial public hospitals in Zhejiang, including 7 general hospitals, 5 specialized hospitals, 2 traditional Chinese medicine hospitals, and 2 integrated traditional Chinese and western medicine hospitals. We conduct an annual survey and determine the sampling quantity based on the business volume of different departments in each public hospital. All medical staff who participated in the survey that year will no longer undergo repeated sampling. A total of 12,405 valid questionnaires were obtained for medical staff. A self-designed medical staff job satisfaction survey questionnaire was used, with a total of 31 related indicators, including 6 sociodemographic factors and 25 hospital factors. The reliability and effectiveness of the questionnaire content are determined through expert consistency evaluation, which can ensure the authority and scientificity of the questionnaire. The consistency test results of the questionnaire indicate that the Cronbach’s Alpha coefficient is 0.944, indicating high reliability of the questionnaire.

### Method of investigation

The outcome variables (medical staff job satisfaction) and explanatory variables of this paper are based on the Likert five level scoring method, with scores of 1 being very dissatisfied, 2 being not very satisfied, 3 being average, 4 being relatively satisfied, and 5 being very satisfied. And further simplify it into two categorical variables: combine “very satisfied” and “relatively satisfied” to “satisfied” (with a value of 1); The other answer combination is ‘dissatisfied’ (value 0). Missing and abnormal values are assigned a value of 99 and removed in subsequent data analysis. The data analysis was completed using SPSS 22.0 and R3.6.1 software.

### Sample quality control

The minimum sample size required first has to be determined before the statistical model is established. The sample size calculation is shown in Formula 1:


(1) $$n=\frac{Z_{\alpha /2}^{2}*p\left(1-p\right)}{\delta^{2}}$$

Where n is the sample size, $Z_{\alpha /2}$ value is 1.96 typically, p is the overall staff job satisfaction rate and δ is the desired level of precision. And then, we assumed 95% confidence and 5% precision. The overall staff job satisfaction in this study is 25.62%. Therefore, the minimum sample size was: $n=\frac{1.96^{2}*0.2562*\left(1-0.2562\right)}{0.05^{2}}\approx 293$ . The number of effective simple in this paper is 12,405, which is far greater than the minimum sample size required.

### Multiple logistic regression

Logistic regression model is one of the supervised algorithms, which adds a sigmoid function to classify based on linear regression and sets a threshold value to map the results to the (0, 1) interval.

Further, when the mapping value is greater than the threshold value, it is classified as 1, and when the mapping value is less than the threshold value, it is classified as 0. In this study, we first conducted a single factor analysis of the explanatory variables. Indeed, the influencing factors with statistical differences (*p* < 0.05) were included in the multiple logistic regression model based on the single factor analysis results. The calculation is shown in Formula 2:


(2) $$\ln\frac{P}{1-P}=\mathrm{Logit}\left(P\right)=\beta 0+\beta 1x1+\cdots +\mathrm{\beta nxn}+\varepsilon$$

The probability prediction formula for employee job satisfaction is shown in Formula 3:


(3) $$P=\frac{1}{1+\exp\left[-\left(\beta_{0}+\beta_{1}x_{1}+\ldots +\beta_{n}x_{n}\right)\right]}$$

Where P and 1−P are the probabilities of overall job satisfaction and dissatisfaction by medical staff; n is the number of independent variables; $\beta_{i}$ presents the regression coefficient of each associated factor; $x_{i}$ present different independent variables and ε is a random interference term.

### Gradient boosting decision tree algorithm

GBDT is an efficient decision tree algorithm that combines weak prediction models to obtain stronger prediction models (45). Specifically, CART regression trees are used to generate weak models by defining loss functions, and then the defined loss function is optimized by pre ordering and adding regular terms to achieve algorithm improvement. The specific construction method of the model is as follows:

Firstly, we construct the medical staff job satisfaction dataset D. As shown in Formula 4:


(4) $$D=\left\{\left(x^{\left(1\right)},y^{\left(1\right)}\right),\left(x^{\left(2\right)},y^{\left(2\right)}\right),\ldots ,\left(x^{\left(n\right)},y^{\left(n\right)}\right)\right\}$$

Where $x^{\left(i\right)}$ and $y^{\left(1\right)}$ are explanatory variables and outcome variables. Training set D and fit it a weak learner model $f_{1}\left(x\right)\text{.}$

Secondly, calculating the negative gradient of the loss function for each sample and generate a new dataset $D^{\prime }$ . As shown in Formulas 5 and 6:


(5) $$R_{K}^{\left(i\right)}=-{\left[\frac{\partial L\left(\;y^{\left(i\right)},\;f\left(x\right)\right)}{\partial f\left(x\right)}\right]}_{f\left(x\right)=f_{k-1}\left(x\right)}$$

(6) $$D^{\prime }=\left\{\left(x^{\left(1\right)},\;R_{K}^{\left(1\right)}\right),\;\left(x^{\left(2\right)},R_{K}^{\left(2\right)}\right),\ldots ,\left(x^{\left(n\right)},\;R_{K}^{\left(n\right)}\right)\right\}$$

Thirdly, we can obtain the regression tree $f_{K}\left(x\right)$ by using the new dataset $D^{\prime }$ . As shown in Formulas 7 and 8:


(7) $$f_{K}\left(x\right)=\overset{M}{\underset{m=1}{\sum }}C_{m}I\left(x^{\left(i\right)}\epsilon \;Q^{\left(m\right)}\right)$$

(8) $$C_{m}=\frac{1}{n}\underset{x^{\left(i\right)}\epsilon \;Q^{\left(m\right)}}{\sum }y^{\left(i\right)}$$

Where M is the node of the leaf of the tree; Q present the total value range of M ; n represents the number of samples per leaf node.

Finally, the optimized model is obtained through K-round iteration. As shown in Formula 9:


(9) $$Ϝ\left(x\right)=\overset{k}{\underset{k=1}{\sum }}f_{K}\left(x\right)=\overset{k}{\underset{k=1}{\sum }}\overset{M}{\underset{m=1}{\sum }}C_{m}I\left(x^{\left(i\right)}\epsilon \;Q^{\left(m\right)}\right)$$

### Naive Bayesian algorithm

Naive Bayesian algorithm is a relatively stable classification algorithm based on Bayesian theorem (46). Firstly, the joint probability distribution of the sample set is trained, and then the output model with the maximum posterior probability is obtained based on the training results. The naive Bayesian algorithm combines *a priori* and *a posteriori* probability, which avoids the subjective bias of using only *a priori* probability and avoids the over fitting phenomenon of using sample information alone (47). Especially when the data set is large, it shows a high accuracy rate. We define staff job satisfaction data training sets $\left(X,Y\right)$ , where each sample $X=\left(x_{1},\;x_{2},\;x_{3},\ldots ,\;x_{n}\right)$ and K categories $Y=\left(y_{1},y_{2},y_{3},\ldots ,y_{k}\right)$ . The calculation process is shown in Formula 10:


(10) $$P\left(y_{k}|x\right)=P\left(x|y_{k}\right)P\left(y_{k}\right)P\left(x\right)=\frac{P\left(x|y_{k}\right)P\left(y_{k}\right)}{\sum_{k}P\left(x|y_{k}\right)P\left(y_{k}\right)}$$

(11) $$P\left(x|y_{k}\right)=P\left(x_{1}|x_{2}|x_{3}|\ldots |x_{n}|y_{k}\right)=\overset{n}{\underset{i=1}{\prod }}P\left(x_{i}|y_{k}\right)$$

(12) $$P\left(y_{k}|x\right)=\frac{P\left(y_{k}\right)\prod_{i=1}^{n}P\left(x_{i}|y_{k}\right)}{\sum_{y}P\left(y_{k}\right)\prod_{i=1}^{n}P\left(x_{i}|y_{k}\right)}$$

Formula 12 is the final form of Formula 10.

Specially, the number of parameters ( $k\overset{n}{\underset{i=1}{\prod }}S_{i}$ ) can be reduced to $\overset{n}{\underset{i=1}{\sum }}S_{i}k$ through Formula (11). Where $P\left(y_{k}|x\right)$ is optimal posterior probability, $P\left(x|y_{k}\right)$ means conditional probability, k is the number of categories and $S_{i}$ means the number of $x_{i}$ .

The final classification model is shown in Formula 13:


(13) $$f\left(x\right)=\mathrm{argma}x_{yk}P\left(y_{k}|x\right)=\mathrm{argmaxP}\left(y_{k}\right)\overset{n}{\underset{i=1}{\prod }}P\left(x_{i}|y_{k}\right)$$

### K-nearest neighbor algorithm

KNN algorithm is also a classification algorithm in supervised learning (48). This algorithm classifies the closest samples in the feature space into one category. At present, Euclidean distance is the most commonly ranging method. The calculation progress of Euclidean distance is as Formula 14:


(14) $$\begin{array}{l} D\left(x,y\right):\sqrt{{\left(x_{1}-y_{1}\right)}^{2}+{\left(x_{2}-y_{2}\right)}^{2}+{\left(x_{3}-y_{3}\right)}^{2}+\ldots +{\left(x_{n}-y_{n}\right)}^{2}}\\ \;=\sqrt{\overset{n}{\underset{i=1}{\sum }}{\left(x_{i}-y_{i}\right)}^{2}}\; \end{array}$$

Generally, a suitable *k* value is selected through cross validation based on the distribution of samples. Then return to the category with the highest frequency of occurrence of the first k points as the optimal prediction classification.

### Random forest algorithm

Random forest model is an excellent bagging ensemble algorithm that fits the optimal multi-classification combination model through comprehensive comparison of random features based on a decision tree. The formula for calculation is as follows:

Firstly, we divide the data set D (Formula 15) into a training set A (70% of the data is used to build the model) and a test set B (30% of the data is used to fit the optimal model).


$$D=\left\{\left(x_{1},y_{1}\right),\left(x_{2},y_{2}\right),\ldots ,\left(x_{m},y_{m}\right)\right\}$$

(15) D=A∪B,A∪B=∅

Secondly, we use training set data A to construct the basic learning algorithm h . As shown in Formula 16. Then, the out-of-bag estimate (*oob*) of was calculated by *B* through Formula (17).


(16) $$T=\left\{h_{1},h_{2},\ldots ,h_{t}\right\}\;,h_{t}=h_{t}\left(x\right)=L\left(A,A_{bs}\right)$$

(17) $$e^{oob}=\frac{1}{\left|A\right|}\underset{\left(x,y\right)\epsilon A}{\sum }∥\left(H^{oob}\neq y\right)$$

Finally, a classification model with the best fit degree is calculated through Formula (18):


(18) $$H^{oob}\left(x\right)=\mathrm{argma}x_{y\in \gamma }\overset{T}{\underset{t=1}{\sum }}I\left(h_{t}\left(x\right)=y\right)$$

Where T is number of base learning algorithms, $A_{bs}$ is sample distribution of training set data, $H^{oob}\left(x\right)$ is the combined classifier model and $I\left(*\right)$ is an indicator function.

In order to further explore the degree of influence between explanatory variables, we calculated the importance of different independent variables through *Gini* coefficient in the model. As shown in Formula 19:


(19) $$GI_{m}=\overset{\left|K\right|}{\underset{k=1}{\sum }}\underset{k\prime \neq k}{\sum }p_{mk}p_{mk\prime }=1-\overset{\left|K\right|}{\underset{k=1}{\sum }}p_{mk}^{2}$$

(20) $$VIM_{jm}^{\left(\mathrm{Gini}\right)}=GI_{m}-GI_{l}-GI_{r}$$

(21) $$VIM_{j}^{\left(\mathrm{Gini}\right)}=\overset{n}{\underset{i=1}{\sum }}VIM_{jm}^{\left(\mathrm{Gini}\right)}$$

Finally, all calculated importance scores are normalized through Formula (22):


(22) $$VIM_{j}=\frac{VIM_{j}}{\sum_{i=1}^{c}VIM_{i}}$$

Where *K* is the number of categories, $p_{mk}$ is the proportion of category *k* in node *m*. $GI_{l}$ and $GI_{r}$ represent the *Gine* coefficient of the two new nodes after branching. And $VIM_{j}$ is the importance score of the *j_th_* characteristic was caculated through Formulas (20, 21).

### Building an optimal evaluation model

This paper incorporates as many mainstream classification and discrimination models as possible. We attempt to ensure the accuracy of the results and calculate the best evaluation model by comparing five models: Multiple logistic regression model, GBDT algorithm, Naive Bayes algorithm, KNN algorithm, and Random forest algorithm.

Generally speaking, the effectiveness of models are comprehensively judged by five indicators: *Accuracy, Classification, Precision, Recall,* and *F1_Score.* Indeed, we visualize the classification effects of different machine learning algorithms by drawing receiver operating characteristic (ROC) curves. And we use AUC (Area Under Curve) to determine the accuracy of the model through Formula (23):


(23) $$AUC=\frac{\sum_{i\in \mathrm{positiveClass}}\mathrm{ran}k_{i}-\frac{M\left(1+M\right)}{2}}{M*N}$$

Where *M* is the number of positive samples; *N* is the number of sub samples.

## Results

### Overall description of the analysis

The results showed that the overall job satisfaction rate of staff in large public hospitals was low (25.62%), while the job satisfaction rate of male staff was significantly higher than that of female staff. In particular, the proportion of female staff among all staff is 72.04%. The reason may be that the daily medical work of public hospitals requires a large number of female nursing staff. At the same time, the proportion of staff with bachelor’s degree or above in this study is 99.61%, with the highest proportion of master’s degree students (42.36%). As a result, medical staff are over 30 years old. Interestingly, almost all medical staff have been assessed with relevant professional titles (95.98%), with primary and lower professional titles accounting for 40.9%. The proportion of years of service between 10 and 15 years is the largest (25.32%). And the compliers accounts for 92%.

### Single factor analysis of medical staff job satisfaction

The analysis results showed that there were significant differences in the job satisfaction of medical staff among sociodemographic factors such as gender, age, educational background, professional title, years of service, Compilers, and almost all hospital factors (*p* < 0.05). As shown in Table 1. Specially, Age, Professional title, Years of service, Interested in work, Time Freedom, and competence for this job are all protective factors for medical staff job satisfaction. Meanwhile, most other variables are associated factors.

**Table 1:** Results of the multivariate analysis.

Variables	Number (%)	Poor (%)	Good (%)	*p* value	OR (95%CI)
Sociodemographic factors					
Gender(B1)				**<0.001**	
male	3,468 (27.96)	2,502 (26.89)	966 (31.16)		1
female	8,937 (72.04)	6,803 (73.11)	2,134 (68.84)		0.813 (0.741, 0.890)
Age(B2)				**<0.001**	
31–40 years old	7,300 (58.82)	4,989 (53.62)	2,311 (74.55)		1
41–50 years old	4,602 (37.10)	3,852 (41.40)	750 (24.19)	**<0.001**	5.51 (3.96, 7.67)
Over 50 years old	503 (4.10)	464 (4.98)	39 (1.26)	**<0.001**	2.32 (1.66, 3.24)
Educational background(B3)				**<0.001**	
High school/technical secondary school and below	47 (0.39)	45 (0.48)	2 (0.06)		1
Undergraduate/Junior College	4,325 (34.86)	4,169 (44.80)	156 (5.03)	**<0.001**	0.07 (0.02, 0.29)
Master’s degree candidate	5,255 (42.36)	3,400 (36.54)	1855 (59.84)	**<0.001**	0.06 (0.05, 0.07)
PhD Candidate	2,778 (22.39)	1,691 (18.17)	1,087 (35.06)	**0.001**	0.85 (0.72, 0.93)
Professional title(B4)					
No	498 (4.02)	332 (3.57)	166 (5.35)		1
primary	4,575 (36.88)	3,303 (35.50)	1,272 (41.03)	**<0.001**	1.75 (1.36, 2.26)
Middle	5,023 (40.49)	3,889 (41.79)	1,134 (36.58)	**0.001**	1.35 (1.12, 1.62)
Deputy Senior	1,548 (12.48)	1,189 (12.78)	359 (11.58)	0.821	1.02 (0.85, 1.23)
senior	761 (6.13)	592 (6.42)	169 (5.45)	0.597	1.06 (0.86, 1.30)
Years of service(B5)				**<0.001**	
2 years and below	1,339 (10.79)	938 (10.08)	401 (12.94)		1
3–5 years	2,896 (23.35)	2049 (22.02)	847 (27.32)	**<0.001**	8.04 (6.38, 1.13)
6–10 years	3,009 (24.26)	2,286 (24.57)	723 (23.32)	**<0.001**	7.77 (6.27, 9.63)
10–15 years	3,142 (25.32)	2,115 (22.73)	1,027 (33.13)	**<0.001**	5.94 (4.79, 7.38)
Over 15 years	2019 (16.28)	1917 (20.60)	102 (3.3)	**<0.001**	9.13 (7.38, 11.29)
Compilers(B6)				**<0.001**	
No	996 (8.00)	579 (6.26)	417 (13.45)		1
Yes	11,409 (92.00)	8,726 (93.74)	2,683 (86.55)		0.43 (0.37, 0.49)
Hospital factors					
Work environment satisfaction(Q1)				**<0.001**	
No	3,851 (31.04)	926 (9.95)	2,925 (94.35)		1
Yes	8,554 (68.96)	8,379 (90.05)	175 (5.65)		0.007 (0.006, 0.008)
Hardware satisfaction(Q2)				**<0.001**	
No	3,884 (31.31)	981 (10.54)	2,903 (93.65)		1
Yes	8,521 (68.69)	8,324 (89.46)	197 (6.35)		0.008 (0.007, 0.009)
Working intensity(Q3)				**<0.001**	
Busy	9,824 (79.19)	8,500 (91.35)	1,324 (42.71)		1
Easy	2,581 (20.81)	805 (8.65)	1776 (57.29)		14.16 (12.80,15.68)
Rewards satisfaction(Q4)				**<0.001**	
No	5,820 (46.92)	3,113 (33.46)	2,707 (87.32)		1
Yes	6,585 (53.08)	6,192 (66.54)	393 (12.68)		0.07 (0.06, 0.08)
Performance evaluation satisfaction(Q5)				**<0.001**	
No	5,118 (41.26)	2,173 (23.35)	2,945 (95.00)		1
Yes	7,287 (58.74)	7,132 (76.65)	155 (5.00)		0.016 (0.014, 0.019)
Satisfaction with vacation arrangements(Q6)				**<0.001**	
No	9,831 (79.25)	7,038 (75.64)	2,793 (90.10)		1
Yes	2,574 (20.75)	2,267 (24.36)	307 (9.90)		0.34 (0.30, 0.39)
Academic atmosphere(Q7)				**<0.001**	
No	4,171 (33.62)	1,257 (13.51)	2,914 (94.00)		1
Yes	8,234 (66.38)	8,048 (86.50)	186 (6.00)		0.01 (0.008, 0.012)
Timeliness of work feedback(Q8)				**<0.001**	
No	3,870 (31.20)	875 (9.40)	2,995 (96.61)		1
Yes	8,535 (68.80)	8,430 (90.60)	105 (3.39)		0.004 (0.003, 0.004)
Democracy(Q9)				**<0.001**	
No	4,325 (34.86)	1,317 (14.15)	3,008 (97.00)		1
Yes	8,080 (65.14)	7,988 (85.85)	92 (3.00)		0.005 (0.004, 0.006)
Value staff opinions(Q10)				**<0.001**	
No	4,459 (35.95)	1,448 (15.56)	3,011 (97.13)		1
Yes	7,946 (64.05)	7,857 (84.44)	89 (2.87)		0.005 (0.004, 0.007)
Get recognition for your work(Q11)				**<0.001**	
No	3,992 (32.18)	955 (10.26)	3,037 (97.97)		1
Yes	8,413 (67.82)	8,350 (89.74)	63 (2.03)		0.002 (0.002, 0.003)
Satisfaction with cultural construction(Q12)				**<0.001**	
No	3,821 (30.80)	842 (9.05)	2,979 (96.10)		1
Yes	8,584 (69.20)	8,463 (90.95)	121 (3.90)		0.004 (0.003, 0.005)
Harmonious relationship with colleagues(Q13)				**<0.001**	
No	3,136 (25.28)	393 (4.22)	2,743 (88.48)		1
Yes	9,269 (74.72)	8,912 (95.78)	357 (11.52)		0.006 (0.005, 0.007)
Work together(Q14)				**<0.001**	
No	2,981 (24.00)	285 (3.06)	2,696 (86.97)		1
Yes	9,424 (76.00)	9,020 (96.94)	404 (13.03)		0.005 (0.004, 0.006)
Colleague communication frequency(Q15)				**<0.001**	
Low	3,723 (30.01)	2,362 (74.32)	136 (1.47)		1
High	9,907 (79.90)	816 (25.68)	9,091 (98.53)		0.011 (0.010, 0.013)
Define the development of the hospital(Q16)				**<0.001**	
No	3,483 (28.08)	621 (6.67)	2,862 (92.32)		1
Yes	8,922 (71.92)	8,684 (93.33)	238 (7.68)		0.006 (0.005,0.007)
Position satisfaction(Q17)				**<0.001**	
No	3,534 (28.49)	651 (7.00)	2,883 (93.00)		1
Yes	8,871 (71.51)	8,654 (93.00)	217 (7.00)		0.006 (0.005, 0.007)
Convenient communication(Q18)				**<0.001**	
No	3,926 (31.65)	971 (10.44)	2,955 (95.32)		1
Yes	8,479 (68.35)	8,334 (89.56)	145 (4.68)		0.006 (0.005, 0.007)
fringe benefits(Q19)				**<0.001**	
No	5,530 (44.58)	2,613 (28.08)	2,917 (94.10)		1
Yes	6,875 (55.42)	6,692 (71.92)	183 (5.90)		0.024 (0.021, 0.029)
Satisfaction with promotion system(Q20)				**<0.001**	
No	5,504 (44.37)	2,606 (28.01)	2,898 (93.48)		1
Yes	6,901 (55.63)	6,699 (71.99)	202 (6.52)		0.027 (0.023, 0.031)
Training system(Q21)				**<0.001**	
No	4,135 (33.33)	1,178 (12.66)	2,957 (95.39)		1
Yes	8,270 (66.67)	8,127 (87.34)	143 (4.61)		0.007 (0.006, 0.008)
Take pride in your hospital(Q22)				0.052	
No	491 (3.96)	350 (3.76)	141 (4.55)		1
Yes	11,914 (96.04)	8,955 (96.24)	2,959 (95.45)		0.820 (0.672, 1.002)
Interested in work(Q23)				**0.001**	
No	4,673 (37.67)	3,595 (38.64)	1,078 (34.77)		1
Yes	7,732 (62.33)	5,710 (61.36)	2022 (665.23)		1.18 (1.09, 1.29)
Time Freedom(Q24)				**<0.001**	
Low	4,370 (35.23)	3,426 (36.82)	944 (30.45)		1
High	8,035 (64.77)	5,879 (63.18)	2,156 (69.55)		1.331 (1.220, 1.452)
Competent for this job(Q25)				**<0.001**	
No	2,969 (31.91)	432 (13.94)	3,401 (27.42)		1
Yes	6,336 (68.09)	2,668 (86.06)	9,004 (72.58)		2.894 (2.591, 3.232)

α =0.05. The meaning is the P-values with significant statistical differences.

### Multiple logistic regression analysis of medical staff job satisfaction

We included independent variables with significant statistical differences in univariate analysis into a multiple logistic regression model. The results showed that the multiple logistic regression results of medical staff job satisfaction are basically consistent with the results of random forest. There is a wide gap between Gender and Educational background. The results showed that almost all hospital factors were closely related to the improvement of medical staff job satisfaction (*p* < 0.05) and were consistent with the results of random forest analysis. In particular, low levels of education are significantly related to medical staff job satisfaction, without the need to achieve the highest level of education, such as Doctors. As shown in Table 2.

**Table 2:** the results of multivariate logistic regression.

Variables	regression coefficient	*p* value	OR (95%CI)
Gender(B1)	−0.551	<0.001	0.576 (0.423, 0.785)
Educational background(B3)		0.005	
High school/technical secondary school and below	−2.485	0.019	0.083 (0.010, 0.662)
Undergraduate/Junior College	−0.783	0.004	0.457 (0.269, 0.779)
Work environment satisfaction(Q1)	−1.569	<0.001	0.208 (0.150, 0.289)
Hardware satisfaction(Q2)	−1.110	<0.001	0.330 (0.239, 0.455)
Rewards satisfaction(Q4)	−0.668	0.010	0.513 (0.308, 0.854)
Performance evaluation satisfaction(Q5)	−1.569	<0.001	0.208 (0.131, 0.331)
Satisfaction with vacation arrangements(Q6)	−0.589	<0.001	0.555 (0.404, 0.761)
Academic atmosphere(Q7)	−0.867	<0.001	0.420 (0.305, 0.579)
Timeliness of work feedback(Q8)	−1.046	<0.001	0.351 (0.246, 0.502)
Democracy(Q9)	−1.153	<0.001	0.316 (0.197, 0.506)
Value staff opinions(Q10)	−0.761	0.003	0.467 (0.283, 0.770)
Get recognition for your work(Q11)	−1.407	<0.001	0.245 (0.161, 0.373)
Satisfaction with cultural construction(Q12)	−0.673	<0.001	0.510 (0.362, 0.719)
Harmonious relationship with colleagues(Q13)	−1.028	<0.001	0.358 (0.225, 0.570)
Work together(Q14)	−0.719	0.009	0.487 (0.285, 0.833)
Colleague communication frequency(Q15)	−0.545	0.003	0.580 (0.407, 0.826)
Define the development of the hospital(Q16)	−0.870	<0.001	0.419 (0.305, 0.577)
Position satisfaction(Q17)	−1.080	<0.001	0.340 (0.246, 0.469)
Convenient communication(Q18)	−0.610	<0.001	0.543 (0.389, 0.759)
fringe benefits(Q19)	−0.981	<0.001	0.375 (0.236, 0.595)
Satisfaction with promotion system(Q20)	−0.705	0.005	0.494 (0.302, 0.808)
Training system(Q21)	−1.033	<0.001	0.356 (0.255, 0.498)
Interested in work(Q23)	1.168	<0.001	3.217 (2.312, 4.476)
Time Freedom(Q24)	1.248	<0.001	3.482 (2.491, 4.867)
Competent for this job(Q25)	0.975	<0.001	2.652 (1.864, 3.774)

### Optimal evaluation model

**Figure 1:**
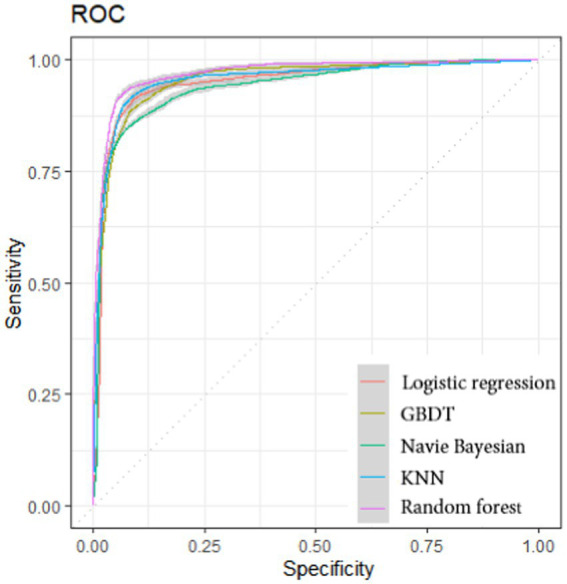
Receiver operating characteristic curves of five classification models.

We calculated the accuracy and ROC curves of different models to compare the robustness of different models. The results show that random forests rank first in *Accuracy* and AUC, with the most accurate prediction effect. Figure 1 shows the ROC curves of the five models, with the results ranked in the order of Random forest (0.9713), KNN (0.9579), GBDT (0.9520), logical regression (0.9478) and Naive Bayesian (0.9378), with the Random forest model performing best.

Table 3 shows that all models have achieved good results. The random forest model is superior to KNN, GBDT, logistic regression model, and naive Bayesian model in accuracy index. The random forest model has the highest evaluation effect and the best performance effect in this study. With good practicality and flexibility, random forest models can not only make high-precision classification decisions, but also calculate the importance of each variable.

**Table 3:** Comparison of evaluation effects of different evaluation models.

Model	Index *p* OR(95%CI)					
	*Accuracy*	*Classification Error*	*Precision*	*Recall*	*F1_Score*	*Auc*
Random forest	0.9344	0.0655	0.9040	0.8967	0.9003	0.9713
Logistics regression	0.9172	0.0827	0.8747	0.8747	0.8747	0.9478
Navie Bayesian	0.8234	0.1765	0.6690	0.9219	0.7753	0.9378
GBDT	0.9083	0.0916	0.8610	0.8617	0.8613	0.9520
KNN	0.9221	0.0778	0.8754	0.8912	0.8832	0.9579

### Importance of different explanatory variables

**Figure 2:**
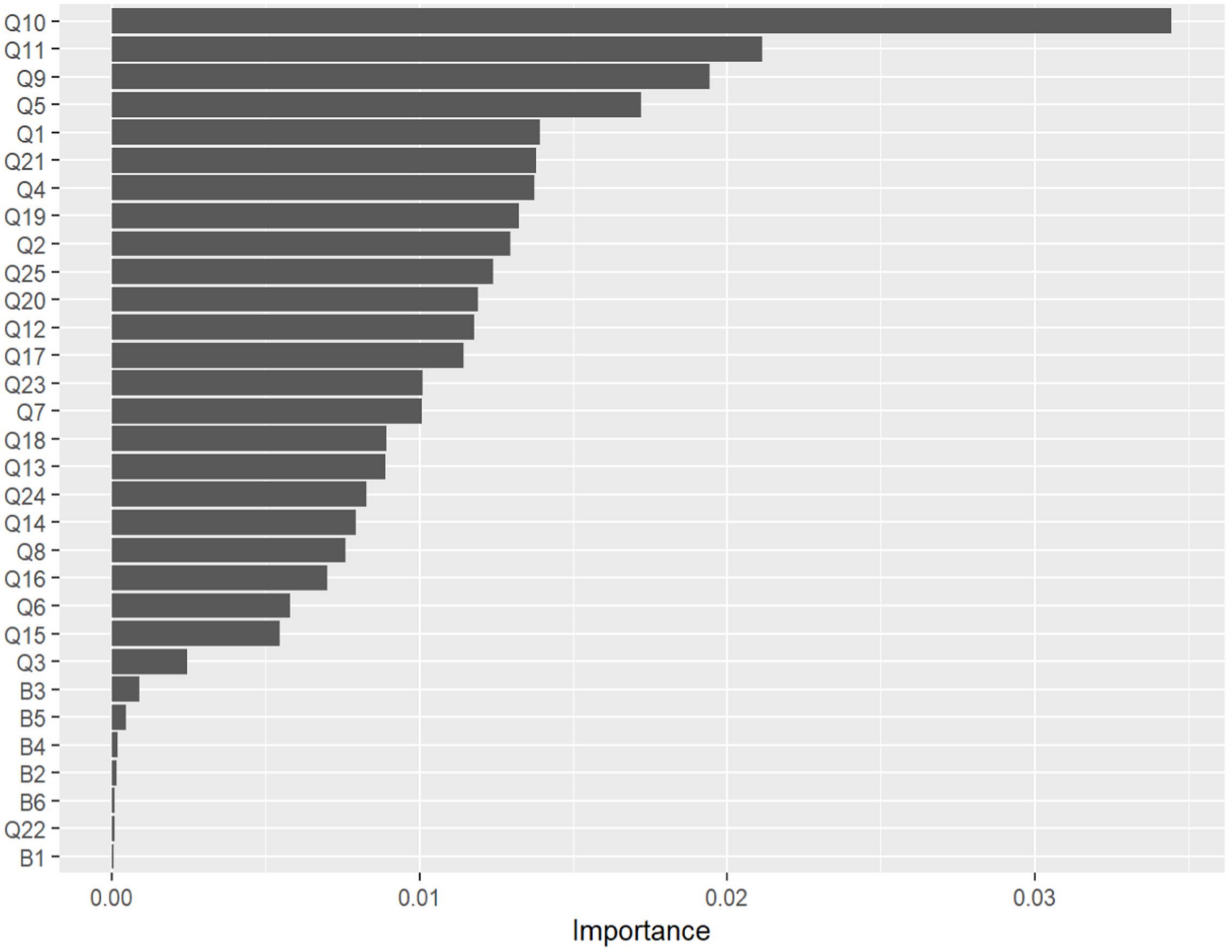
Importance ranking chart of influencing factors.

We plotted the importance ranking diagram of all explanatory variables through a random forest algorithm. As shown in Figure 2. Value staff opinions (Q10), Get recognition for your work (Q11), Democracy (Q9) and Performance evaluation satisfaction (Q5) rank in the top 4 among all variables.

### Conclusion and discussion

In this study, we evaluated the effectiveness of five widely used models, including logistic regression, Random forest, Naive Bayesian, GBDT, and KNN. The results showed that the random forest model ranked first in accuracy and roc curve in this study. Therefore, we constructed an optimal evaluation model and explored the key variables that affect medical staff job satisfaction in Chinese public hospitals. Further, we can adopt the most appropriate strategies to improve the challenges faced by medical staff. This study shows a weak association between sociodemographic factors such as gender, age, educational background, and medical staff job satisfaction, which is consistent with previous studies (49). This further confirms that although factors such as age and educational background are the key to entering a hospital job, the key to ensuring high job satisfaction among medical staff lies more in the job itself. Interestingly, Compilers is not a key variable in staff job satisfaction in large public hospitals. Value staff opinions (Q10), Get recognition for your work (Q11), Democracy(Q9) and Performance evaluation satisfaction(Q5) are the four most important key factors that affect the satisfaction of medical staff, which provide a neglected perspective for improving the enthusiasm of medical staff in past research. This may be an effective way to improve medical staff satisfaction by weakening the direct authority of organizational leaders and paying more attention to the medical services provided to patients.

The evaluation and prediction of staff in Chinese public hospitals is very important due to undertaking major diagnostic treatment and the promotion and application of the most advanced medical technology (50). Furthermore, large public hospitals are the leaders of medical service complexes within a certain geographical range (51). This paper make some contribution from the following aspects. Firstly, the rapid changes in the disease spectrum and the rapidly increasing demand for individual medical services not only directly increase the difficulty of medical services, but indirectly increasing the challenges faced by medical staff. Few studies have explored the key factors from the perspective of medical staff, and we have conducted in-depth analysis of this. A large number of studies have confirmed the positive indirect role played by medical workers inpatient rehabilitation. Meanwhile, job satisfaction, occupational well-being and harmonious doctor-patient relationships all positively affect the work quality of medical staff (52). In addition, this is an effective measure to promote medical staff to actively provide high-quality services to effectively identify key variables that affect medical staff job satisfaction through the optimal evaluation model.

Although few studies have explored the best evaluation model for medical staff job satisfaction, some studies do emphasize that appropriate analytical models can increase the accuracy of research results (53). Therefore, understanding the actual needs of public medical staff will significantly improve the doctor-patient ecological environment and maximize medical staff job satisfaction with minimal resources.

First of all, value staff opinions is the most important influencing factor of staff job satisfaction. Ample studies believed that strengthening the importance attached by hospital leaders to staff is beneficial to improving the job satisfaction of medical staff (54–56). According to social exchange theory, when employees or individuals feel support from their organizations, they have a strong sense of obligation and belonging (57). This sense of obligation and belonging can be externalized into corresponding social behaviors, including actively providing assistance and consciously promoting work enthusiasm (58). Medical staff more need the care and support of organizational leaders due to the more severe work pressure in the post epidemic era (59). Only staff with high job satisfaction can meet the needs of patients to the maximum extent, and all patient-centered service concepts can be realized (60, 61).

Secondly, get recognition for your work ranks second among all variables that affect staff job satisfaction. Obtaining recognition from others allows individuals to feel their own value in team work. Goal setting theory believes that people will work harder and engage in achieving their goals, as a positive feedback that can promote better development of personnel (62). Especially in the field of health, the work of medical staff requires more recognition and attention from the organization.

The leadership of public hospitals often ignores the positive affirmation of medical staff and unilaterally emphasizes the economic benefits of hospitals. And medical staff often have a reduced sense of self-worth and are discouraged from working due to a lack of leadership attention. Therefore, multi-point practice policy of China for doctors can not only improve their economic income and reputation, but also maximize their self-worth (63). In addition, it is recommended that informal democratic life meetings be held frequently to strengthen the relationship between hospital employees and their co organizational leaders. Increasing recognition of self-work and achieving self-worth through praise and self-praise are potentially effective strategies.

Thirdly, democracy is the third key variable that affects medical staff job satisfaction. Self-determination theory believes that individuals pursue autonomy and control after meeting their basic needs (64). Equity and democracy can effectively promote the sustainable development of public health (65). China has implemented a large number of health policy reforms, including centralized drug procurement policies and DRG(s) payment policies, which have effectively curbed the bureaucracy and corruption in public hospitals over the past decade. Medical staff have played a key role in epidemic prevention and control, benefiting from a high degree of democracy in public hospitals. Trust in institutions and democracy has also been further validated in vaccination (66). Therefore, effective strategies should continue to be adopted to maintain the democratization of public hospitals, including empowering medical staff to make decisions in the face of major decisions regarding hospital development and the interests of employee groups. The top three satisfaction influencing factors in this study are significantly related to hospital organizational leadership. Hence, democratic centralism is an effective measure that can not only ensure the rationality of decision-making but also effectively avoid the personal style of leaders in hospital organization and management (67).

Fourth, performance evaluation satisfaction is also crucial for hospital staff job satisfaction. The reform of the personnel and salary distribution system in public hospitals has been one of the core elements of health care reform over the past 10 years. Relevant research has confirmed that income distribution is one of the most important aspects of hospital performance evaluation for medical staff (68). At present, there are still problems in the performance evaluation of public hospitals in China, such as emphasizing economic benefits, unreasonable indicator settings, and imperfect allocation methods (69). A series of health policies have significantly reduced hospital income while reducing the economic burden on patients, resulting in a decrease in the income of medical staff (70). The reason may be that different departments of the hospital use unified assessment indicators (71).

In addition, the current staff incentive in public hospitals in China mainly focuses on simple and convenient salary incentives, which are also prone to problems such as generalization of incentive effects and excessive utilitarian orientation. Specifically, the large income gap among different staff is due to the large difference in the distribution coefficient of professional titles (72, 73). Therefore, the performance evaluation of public hospitals should focus on improving medical quality, promoting hospital development, and enhancing social benefits. This is an effective measure to promote the development of conscience in public hospitals to improve a more scientific performance and evaluation indicator system.

Finally, machine learning algorithms provide a new research direction for research on hospital staff job satisfaction. Meanwhile, a good working environment can create a better working atmosphere and stimulate the work creativity of medical staff. This study attempts to fit the best discriminant model for medical staff job satisfaction from the perspective of health human resources. Compared with traditional linear algorithms and other machine learning algorithms, random forests have the highest accuracy and best prediction results. Therefore, we suggest using random forest algorithm to explore relevant studies on the factors affecting job satisfaction in future.

## Strengths and limitations of this study

The strengths are the cross-sectional survey of a large sample for three consecutive years, the department service volume based sample, the most appropriate discriminant model and potential applicability of our findings to many settings, since high-quality healthcare human resources have long been scarce, especially in the post pandemic era. The unique aspect of this study lies in its design, which includes panel data for almost all types of medical staff in public hospitals and optimal Discriminant Model for Similar Studies. The main limitation is that the investigation was forced to be interrupted after 3 years. Our investigation after 2020 has to stop because of the global epidemic of COVID-19, and it is difficult to recover to the pre epidemic level. In addition, China’s healthcare reform has affected the personnel structure and internal management of public hospitals to varying degrees, which may make precise measurements difficult. Moreover, the same strategy may not apply to medical personnel in all regions due to differences in economic levels and educational resources in different regions of China.

## Data availability statement

The original contributions presented in the study are included in the article/supplementary material, further inquiries can be directed to the corresponding authors.

## Ethics statement

This study was approved and supported by the Zhejiang Provincial Health Commission, and the investigation was conducted after obtaining the consent and support of the relevant heads of 16 hospitals. All participating medical staff signed an informed consent form before filling out the questionnaire. This job satisfaction survey is anonymous and the content filled in is completely confidential.

## Author contributions

CL: Conceptualization, Methodology, Writing – original draft. XM: Conceptualization, Data curation, Writing – review & editing.
